# RNF7 inhibits apoptosis and sunitinib sensitivity and promotes glycolysis in renal cell carcinoma via the SOCS1/JAK/STAT3 feedback loop

**DOI:** 10.1186/s11658-022-00337-5

**Published:** 2022-05-13

**Authors:** Chengwu Xiao, Wei Zhang, Meimian Hua, Huan Chen, Bin Yang, Ye Wang, Qing Yang

**Affiliations:** Department of Urology, Changhai Hospital, Naval Medical University, No. 168 Changhai Road, Yangpu, Shanghai, 200433 China

**Keywords:** Apoptosis, Clear cell renal cell carcinoma, Glycolysis, JAK/STAT3, RNF7, SOCS1

## Abstract

**Background:**

RING finger protein 7 (RNF7) is a highly conserved protein that functions as an E3 ubiquitin ligase. RNF7 overexpression is indicated in multiple human cancers, but its role in renal cell carcinoma (RCC) and the mechanisms underlying how it regulates the initiation and progression of RCC have not been explored.

**Methods:**

Bioinformatics analysis, quantitative reverse-transcription polymerase chain reaction (RT-PCR), and Western blot were conducted to determine the expression of RNF7 in RCC tissues and cell lines. Knockdown and overexpression experiments were performed to examine the effects of RNF7 on cell viability, apoptosis, and glycolysis in vitro and on tumor growth in nude mice in vivo.

**Results:**

The elevated RNF7 expression in tumor tissues of patients with RCC was correlated with poor survival. RNF7 overexpression inhibited apoptosis and promoted glycolysis in vitro and increased tumor growth in vivo by activating the JAK/STAT3 signaling pathway by ubiquitination of SOCS1. Moreover, RNF7 overexpression affected the sensitivity of RCC cells to sunitinib. Finally, STAT3 activation was necessary for transcriptional induction of RNF7.

**Conclusion:**

These results demonstrate that RNF7 inhibited apoptosis, promoted glycolysis, and inhibited sunitinib sensitivity in RCC cells via ubiquitination of SOCS1, thus activating STAT3 signaling. These suggest the potential for targeting the RNF7-SOCS1/JAK/STAT3 pathway for RCC treatment.

**Supplementary Information:**

The online version contains supplementary material available at 10.1186/s11658-022-00337-5.

## Background

Renal cell carcinoma (RCC) originates within the renal cortex and is the most widespread type of kidney cancer in adults. Clear cell RCC (ccRCC) is derived from renal tubular epithelial cells and is the most prevalent RCC subtype, accounting for 80% of cases [1]. The risk factors for ccRCC include both genetic and acquired risk factors. A frequent genetic alteration in ccRCC is the loss of the short arm of chromosome 3 (loss of 3p), which is observed in approximately 95% of ccRCC cases [2]. Several genes involved in the pathogenesis of ccRCC have been reported, including von Hippel–Lindau (*VHL*) [3] and protein polybromo-1 [4]. Inactivation of *VHL* leads to elevated levels of transcription factor hypoxia-inducible factor (HIF)-1α [5] and subsequent upregulation of vascular endothelial growth factor (VEGF) and platelet-derived growth factor (PDGF) expression [6, 7], promoting angiogenesis. Thus, the expression of these proteins has been used as biomarkers of ccRCC.

The prognosis of patients with ccRCC is greatly affected by tumor progression and degree of tumor dissemination. Early-stage ccRCC can be removed by resection, whereas metastatic ccRCC is more likely to result in mortality than early-stage ccRCC and requires targeted therapy. Sunitinib, a tyrosine kinase inhibitor, has been widely used for ccRCC treatment, and it improve the condition of patients with metastatic ccRCC [8]. Thus, it has become a front-line therapy for RCC [9]. Sunitinib displays potent antitumor and antiangiogenic activities by inhibiting VEGF receptors and tyrosine kinases, including PDGF and c-kit receptors [10, 11]. Whether used alone or in combination with classical chemotherapy [12], sunitinib has a high level of efficacy and acceptable tolerability in metastatic RCC [13], indicating its potential for RCC treatment.

RING finger protein-7 (RNF7) is a highly conserved protein that has been implicated as a part of the SCF (SKP1-CUl1-F-box protein) E3 ubiquitin complex [14], which mediates the ubiquitination and proteasomal degradation of target proteins [15]. E3 ubiquitin ligases play essential roles in regulating many biological processes, and accumulating evidence suggests that E3 ubiquitin ligases are often upregulated in human cancers that are associated with chemoresistance and poor clinical prognosis [16]. For instance, it was reported that RNF7 is overexpressed in prostate [17] and lung [18] cancers. However, whether RNF7 plays a role in ccRCC and the molecular mechanisms involved have not been explored.

The signal transducer and activator of transcription 3 (STAT3) are members of the STAT family of proteins that have important roles in relaying signals from activated cytokines and growth factor receptors in the plasma membrane to the nucleus, where they regulate gene transcription [19]. STAT3 regulates the transcription of genes involved in various functions, including viability, apoptosis, metastasis, and immune responses [20]. STAT3 plays central roles in the development, maintenance, and progression of several human cancers [21–23], and STAT3 activation is associated with poor cancer prognosis [22], making this pathway an attractive drug target. Interestingly, sunitinib promotes RCC apoptosis and growth arrest, and it coincides with the inhibition of STAT3 activity [24]. Importantly, a reduced STAT3 activity augments the antitumor effects of sunitinib, whereas the constitutive activation of a STAT3 mutant rescues tumor cell death [24], suggesting that STAT3 activation is a crucial downstream mediator that executes sunitinib’s effects. However, the mechanisms involved here have not been elucidated.

SOCS1, a member of suppressors of cytokine signaling (SOCS) family protein, is a negative feedback regulator of the JAK-STAT signaling pathway [25]. Abnormal expression of SOCS1 is involved in the occurrence and progression of human cancers [25, 26]. In the present study, the RNF7-SOCS1/JAK/STAT3 pathway was identified, and its functional importance in RCC progression was demonstrated. Moreover, an important role of RNF7 in modulating sunitinib sensitivity in RCC cells was highlighted, suggesting a potential therapeutic avenue for RCC.

## Methods

### Bioinformatics analysis

The messenger RNA (mRNA) expression levels of RNF7 and The Cancer Genome Atlas Kidney Renal Clear Cell Carcinoma (TCGA-KIRC) clinical data were downloaded from the TCGA data portal (https://tcga-data.nci.nih.gov/tcga/). Patients with corresponding gene expressions were included in this study, while those with missing overall survival data were excluded. The gene functions were verified via a screening standard, nominal *P* < 0.05, using gene set enrichment analysis (GSEA) software (https://software.broadinstitute.org/gsea/index.jsp) [27]. Gene sets with a distinct peak at the beginning or end of a ranked list are generally the most interesting. For this process, significant *P*-values calculated by 1000 permutations determined whether the genes were enriched or not.

### Clinical samples

A total of 178 patients with ccRCC who underwent nephrectomy at Changhai Hospital between October 2017 and June 2021 were included in this study and divided into two cohorts. The inclusion criteria for these two cohorts included those with clinical and imaging diagnosis of ccRCC, aged 20–86 years old, and who underwent nephrectomy at Changhai Hospital. The exclusion criteria for these two cohorts included those with missing imaging data; patients with severe liver and kidney disease, cardiovascular disease, or blood disease; and patients who received radiochemotherapy before nephrectomy. The tissue samples corrected from 20 patients with ccRCC included in cohort 1 consisted of 20 fresh pairs of ccRCC and adjacent nontumor tissues, while those corrected from 158 patients with ccRCC included in cohort 2 consisted of 158 formalin-fixed paraffin-embedded ccRCC and 20 adjacent nontumor tissues. The distance between the ccRCC samples and adjacent nontumor tissue samples was ≥ 2 cm. The study was carried out according to the provisions of the Declaration of Helsinki of 1975. Written informed consent was obtained from all patients. This study was approved by the Ethics Committee of Changhai Hospital (approval no. CHEC2021-191).

### Immunohistochemistry (IHC)

Paraffin-embedded tissue sections of primary ccRCC and adjacent nontumor tissues were prepared for IHC staining. The tissues were fixed with 4% paraformaldehyde, embedded in paraffin, and subjected to standard dewaxing and rehydration. The sections were incubated in citric acid buffer (pH 6.0) for 15 min for antigen retrieval, followed by incubation for 10 min with 3% H_2_O_2_ solution to inactivate endogenous enzymatic activities. Then, the sections were incubated with anti-RNF7 (ab181986; 1:50; Abcam) and anti-SOCS1 (ab9870; 1:100; Abcam) primary antibodies for 1 h at 25 °C followed by using horseradish peroxidase (HRP)-conjugated second antibody (KIT-9903; 1:1,000; Maxim Biotech, Inc., Rockville, MD, USA) for 30 min at 20 °C. After washing three times with phosphate-buffered saline (PBS), peroxidase activity was visualized using 3,3′-diaminobenzidine (DAB; OriGene Technologies, Inc., Rockville, MD, USA) at 25 °C for 10 s. Then, the sections were counterstained with hematoxylin (OriGene Technologies, Inc.) at room temperature for 3 min. IHC analysis was performed by two pathologists blinded to the pathological and clinical characteristics of these patients. The proportion of positively stained tumor cells was determined. Patients with a proportion of positively stained tumor cells greater than 25% or less than 25% have high or low expression, respectively.

### Cell culture and transfection

Human RCC cell lines [786-O (cat. no. CRL-1932), A498 (cat. no. HTB-44), ACHN (cat. no. CRL-1611), Caki-1 (cat. no. HTB-46), and Caki-2 (cat. no. HTB-47)] and human proximal tubular HK-2 cells (cat. no. CRL-2190) were acquired from the American Type Culture Collection (Manassas, VA, USA), tested, and authenticated by short tandem repeat-based assays. All cell lines were authenticated. Complete policies and requirements were available in the instructions to authors. The cells were replaced from frozen stocks after a maximum of 12 passages or 3 months of continuous culture. The cell lines were periodically confirmed negative for mycoplasma contamination using polymerase chain reaction (PCR) assays. The cells were cultured in Roswell Park Memorial Institute (RPMI)-1640 media (Life Technologies, Carlsbad, CA, USA) containing 10% fetal bovine serum and 1% penicillin/streptomycin (Life Technologies) in an atmosphere of 5% CO_2_ and 95% air at 37 °C. Sunitinib-resistant Caki-1 and ACHN cell lines were generated in stepwise manner by exposing parental cells to increasing doses of sunitinib (1, 5, 10, and 20 µM). Surviving cells were subsequently maintained in a conditioned medium containing 1 µM sunitinib (Sigma-Aldrich Co., St Louis, MO, USA) to retain drug-resistant phenotypes. Parental controls were performed in Caki-1 and ACHN cells with similar passage numbers.

For overexpression studies, the coding sequences of RNF7 or SOCS1 were synthesized and cloned into a pLVX-Puro lentivirus plasmid (Clontech, USA), respectively. An empty pLVX-Puro lentivirus plasmid was regarded as a vector control. RNA interference sequence targeting RNF7 (NM_014245.5) was designed and synthesized by Obio Technology Company and cloned into linearized pLKO.1 lentivirus plasmids (Addgene, USA). Short hairpin RNA (shRNA)-1 (sequence, 5′-CCGGTCGTGGAAGACGGAGAGGAACTCGAGTTCCTCTCCGTCTTCCACGTTTTTG-3′) target position 53-71; shRNA-2 (sequence, 5′-CCGGTACAAGATGTTCTCCCTCAACTCGAGTTGAGGGAGAACATCTTGTTTTTTG-3′) target position 124-142; shRNA-3 (sequence, 5′-CCGGTGGTCCAAAGAATCGGCAAACTCGAGTTTGCCGATTCTTTGGACCTTTTTG-3′) target position 365-383. A nontargeting sequence (5′-CCGGTGAGGCGAGAGCGATAGGAACTCGAGTTCCTATCGCTCTCGCCTC TTTTTG-3′) was inserted into the pLKO.1 lentivirus plasmid as an sh-negative control (NC). Recombinant plasmids and psPAX2 and pMD2G packaging plasmids were transfected into 293 T cells using Lipofectamine 2000 (Invitrogen) for 6 h at 37 °C. After 48 h of transfection, the recombinant lentivirus in the cell supernatant was collected by centrifugation at 5000 × *g* for 5 min at 25 °C, and purification and titration of the recombinant lentivirus were performed, as described previously [28]. The cells were infected with recombinant lentivirus-transducing units at multiplicity of infection of 20 in presence of 8 μg/mL polybrene (Sigma-Aldrich; Merck KGaA) for 24 h at 37 °C. Stable cells were selected with puromycin (3 μg/mL; Thermo Fisher Scientific, Inc.) for four more days and used for subsequent experiments.

### Cell counting kit-8 (CCK-8) assay

ACHN, Caki-1, and Caki-2 cells (3 × 10^3^ cells/well) were cultured in 96-well plates overnight at 37 °C. Caki-1 and Caki-2 cells were transduced with an RNF7-silencing vector. Similarly, ACHN cells were transduced with vectors overexpressing RNF7 and/or SOCS1. After 0, 24, 48, and 72 h of treatment, 10 µL CCK-8 solution was added to each well for 1 h, following the manufacturer’s protocol. Cell viability was measured using a microplate reader by optical density (OD) at 450 nm.

### Cell apoptosis assay

ACHN, Caki-1, and Caki-2 cells (5 × 10^5^ cells/well) were grown in six-well plates until the cells reached 50% confluence. Caki-1 and Caki-2 cells transduced with RNF7-silencing vectors were treated with JAK/STAT inhibitor AG490 (10 µM) and/or sunitinib (1 μM), whereas ACHN cells transduced with RNF7- and/or SOCS1-overexpressing vectors were treated with AG490 (10 µM) and/or sunitinib (1 μM). Cell apoptosis was assessed by flow cytometry at 48 h after the treatment. Briefly, the cells were incubated with 5 µL recombinant annexin V labeled with fluorescein isothiocyanate (annexin V-FITC) for 15 min in the dark at 4 °C and then with 5 µL propidium iodide (PI; Beyotime Institute of Biotechnology) for another 15 min. Apoptosis was examined using a flow cytometer (BD Biosciences, San Jose, CA, USA).

### Estimation of glycolysis

ACHN, Caki-1, and Caki-2 cells (5 × 10^4^ cells/well) were grown in 24-well plates and maintained at 37 °C for 2 days. Caki-1 and Caki-2 cells were transduced with RNF7-silencing vectors, whereas ACHN cells transduced with RNF7- and/or SOCS1-overexpressing vectors were treated with or without AG490 (10 µM). At 48 h after treatment, glycolysis was estimated by extracellular acidification rate (ECAR), as described previously [29]. The evaluation was conducted using a Seahorse XF24 extracellular flux analyzer.

### Measurement of lactate

ACHN, Caki-1, and Caki-2 cells (5 × 10^5^ cells/well) were grown in six-well plates and maintained at 37 °C for 1 day. Caki-1 and Caki-2 cells were transduced with an RNF7-silencing vector, whereas ACHN cells transduced with RNF7- and/or SOCS1-overexpressing vectors were treated with or without AG490 (10 µM). At 48 h after treatment, the lactate release from the cells was determined using a lactic acid assay kit (Nanjing Jiancheng Bioengineering Institute, China), following the manufacturer’s instructions.

### Intracellular ATP content determination

ACHN, Caki-1, and Caki-2 cells (5 × 10^5^ cells/well) were cultured in six-well plates at 37 °C for 1 day. Caki-1 and Caki-2 cells with RNF7 knockdown and ACHN cells with RNF7 and/or SOCS1 overexpression were treated with or without AG490 (10 µM). At 48 h after the treatment, the intracellular adenosine triphosphate (ATP) content was determined using a commercial kit (Nanjing Jiancheng Bioengineering Institute), following the manufacturer’s instructions.

### Immunofluorescence staining

The cells were washed with PBS and fixed in 4% paraformaldehyde for 30 min at room temperature. PBS with Tween-20 and 5% bovine serum albumin (BSA; Cell Signaling Technology, Inc., Danvers, MA, USA) was used to block the washed cells for 30 min at 37 °C. The cells were incubated with anti-RNF7 (ab181986; Abcam) or anti-SOCS1 (ab9870; Abcam) antibodies overnight at 4 °C and subsequently stained with Alexa Fluor 555-labeled (A0453; Beyotime, Shanghai, China) or Alexa Fluor 488-labeled (ab150129; Abcam) secondary antibodies at 37 °C for 30 min in the dark. 4′,6-Diamidino-2-phenylindole (DAPI, C1006; Beyotime Institute of Biotechnology, Haimen, China) was used to stain the nucleus for 15 min at room temperature. Immunopositive cells were observed using a fluorescent microscope (Nikon Corporation, Tokyo, Japan).

### Co-immunoprecipitation

Cell lysates were extracted with radioimmunoprecipitation assay (RIPA) buffer (containing 1 mM dithiothreitol (DTT), 100 mmol/l NaCl, and 1 mM MgCl_2_) and protease inhibitor cocktails (Bimake.com). The lysates were centrifuged at 12,000 × *g* for 10 min at 4 °C. The total cell lysates were used for immunoprecipitation with anti-RNF7 (ab34872; 1:50; Abcam), anti-SOCS1 (PA1-29533; 1:100; Thermo Fisher Scientific), or normal immunoglobulin G (IgG) antibodies (sc-2027; Santa Cruz Biotechnology, Inc.), followed by protein A/G PLUS-Agarose beads (sc-2003; Santa Cruz Biotechnology, Inc.), at 4 °C for 2 h. Immunocomplexes were washed three times in lysis buffer and subjected to Western blotting using anti-RNF7 (ab181986; 1:1000; Abcam), anti-SOCS1 (ab62584; 1:1000; Abcam), and anti-ubiquitin (ab134953; 1:1000; Abcam) antibodies.

### Plasmid generation and dual-luciferase assay

The promoter region of RNF7 was inserted into a luciferase reporter plasmid pGL3-basic (Promega Corporation). For reporter assay, the cells were treated with AG490 and transfected with pGL3-basic-RNF7 promoter and pRL-TK vector (Promega Corporation) expressing a *Renilla* luciferase for normalization at a ratio of 2:2:1 using a Lipofectamine 2000 reagent (Invitrogen; Thermo Fisher Scientific, Inc.). A dual-luciferase reporter assay system (Promega Corporation) was used to measure the luciferase activity at 48 h post-transfection.

### Chromatin immunoprecipitation (ChIP) assay

ChIP assay was performed using an EZ-ChIP kit (Upstate Biotechnology), following the manufacturer’s manual. Briefly, cells with or without an RNF7 mutant (potential STAT3 binding site mutated by the transversion of internal nucleotides, which was performed as previously described) [30, 31] were cross-linked in 1% formaldehyde, and the DNA was sonicated into a size range of 200–1000 base pairs using a Bioruptor sonicator (Diagenode) for five cycles of 3 s on and 3 s off. The extracts were precleared in BSA-blocked protein A/G beads and incubated with anti-STAT3 (Abcam) or control IgG antibodies overnight at 4 °C. After being washed, the DNA was eluted and reverse-cross-linked overnight at 65 °C; then, it was purified and amplified by PCR using RNF7 primers (F, 5′-ACCATCACCTAGTGGTGGA-3′ and R, 5′-ACATGTGATGGTGGGCTAG-3′).

### Quantitative reverse transcriptase-PCR (RT-PCR)

The total RNA was extracted using TRIzol reagent (Life Technologies, Inc., Waltham, MA, USA). Complementary DNA (cDNA) synthesis was performed using a PrimeScript kit (Takara Biotechnology, Dalian, China), following the manufacturer’s protocol. PCR was conducted using SYBR Green PCR master mix (Applied Biosystems, Foster, CA, USA) on an ABI 9700 real-time PCR system (Applied Biosystem). The following primers were used: RNF7-F: 5′-TGCACTCACCTCACTGTTC-3′, RNF7-R: 5′-CACCTGTAATCCCAGCTACTC-3′; SOCS1-F: 5′-CACGCACTTCCGCACATTCC-3′, SOCS1-R: 5′-GCTGCCATCCAGGTGAAAGC-3′; and GAPDH-F: 5′-AATCCCATCACCATCTTC-3′, GAPDH-R: 5′-AGGCTGTTGTCATACTTC-3′. The 2^−ΔΔCT^ method was used to calculate the fold changes in the mRNA expression levels of RNF7 and SOCS1.

### Western blot analysis

Protein lysates were harvested using RIPA lysis buffer with freshly added protease inhibitor cocktail (Sigma, St. Louis, MO, USA). Proteins were separated by sodium dodecyl sulfate (SDS)-polyacrylamide gel electrophoresis (PAGE) and transferred onto nitrocellulose membranes (Millipore, Bedford, USA), which were then blocked with 5% skim milk and incubated with RNF7 (ab181986; 1:5000), SOCS1 (ab62584; 1:600), HK2 (ab227198; 1:8000), STAT3 (ab119352; 1:2000), p-STAT3 (ab76315; 1:5000; all from Abcam), GLUT1 (orb157188; 1:2000; Biorbyt, St Louis, MO, USA), cleaved PARP1 (#5625; 1:1000), cleaved caspase-3 (#9661; 1:1000), and GAPDH (#5174; 1:2000; all from Cell Signaling Technology, Danvers, MA, USA) primary antibodies and HRP-conjugated secondary antibodies (A0208, A0181; 1:1000; Beyotime). Protein bands were visualized using an enhanced chemiluminescence system (Bio-Rad, Richmond, CA, USA). Protein levels were normalized to those of glyceraldehyde 3-phosphate dehydrogenase (GAPDH).

### Generation of stable cell lines and xenograft study

Four- to five-week-old male nude mice were purchased from Shanghai SLAC Laboratory Animal Co., Ltd. (Shanghai, China). The care of laboratory animals and animal experimentation were performed in accordance with animal ethics guidelines and approved protocols. All animal studies were approved by the Animal Ethics Committee of Changhai Hospital (approval no. CHEC2021-191). Twelve mice were randomly divided into two groups to construct a xenograft model. Caki-1 cells (5 × 10^6^) transduced with an RNF7-silencing vector or NC vector were suspended in serum-free RPMI-1640 media and subcutaneously injected into the armpits of each mouse. After 33 days, the mice were anesthetized by inhalation with 3% isoflurane and sacrificed by cervical dislocation. Then, tumor xenografts were collected, photographed, and weighed, and terminal deoxynucleotidyl transferase dUTP nick end-labeling (TUNEL) (Roche, Indianapolis, IN, USA) analysis was performed by a pathologist blinded to group and experiment data (*n* = 6 per group). Another 20 mice were randomly divided into four groups to construct a xenograft model in the absence or presence of sunitinib treatment. ACHN cells (5 × 10^6^) transduced with an RNF7-overexpressing vector or blank vector were suspended in serum-free RPMI-1640 media and were subcutaneously injected into the armpits of each mouse. From day 12, each mouse was intraperitoneally injected with 20 mg/kg sunitinib (Selleck Chemicals LLC, Houston, TX, USA) or vehicle (DMSO; Selleck Chemicals LLC) every other day. The tumor xenografts were collected, photographed, and weighed on day 33. TUNEL analysis was performed as mentioned above (*n* = 5 per group). The animals were anesthetized by inhalation with 3% isoflurane and sacrificed by cervical dislocation on day 33.

### Statistical analysis

Three independent replicates were performed for all assays. Quantitative data are expressed as mean ± standard deviation (SD). Statistical analysis was performed using GraphPad Prism 8.4.2 (GraphPad Software, USA). Two-tailed unpaired or paired Student’s *t*-test was used to compare the differences between the two groups. One-way analysis of variance (ANOVA), followed by Tukey’s post-multiple test, was used to compare the differences between multiple groups. *P* < 0.05 was considered statistically significant. The Kaplan–Meier method and Cox’s proportional hazards regression model were used to calculate the overall survival, and differences between groups were analyzed by log-rank test.

## Results

### RNF7 expression is upregulated in ccRCC and is correlated with poor patient survival

**Fig. 1:**
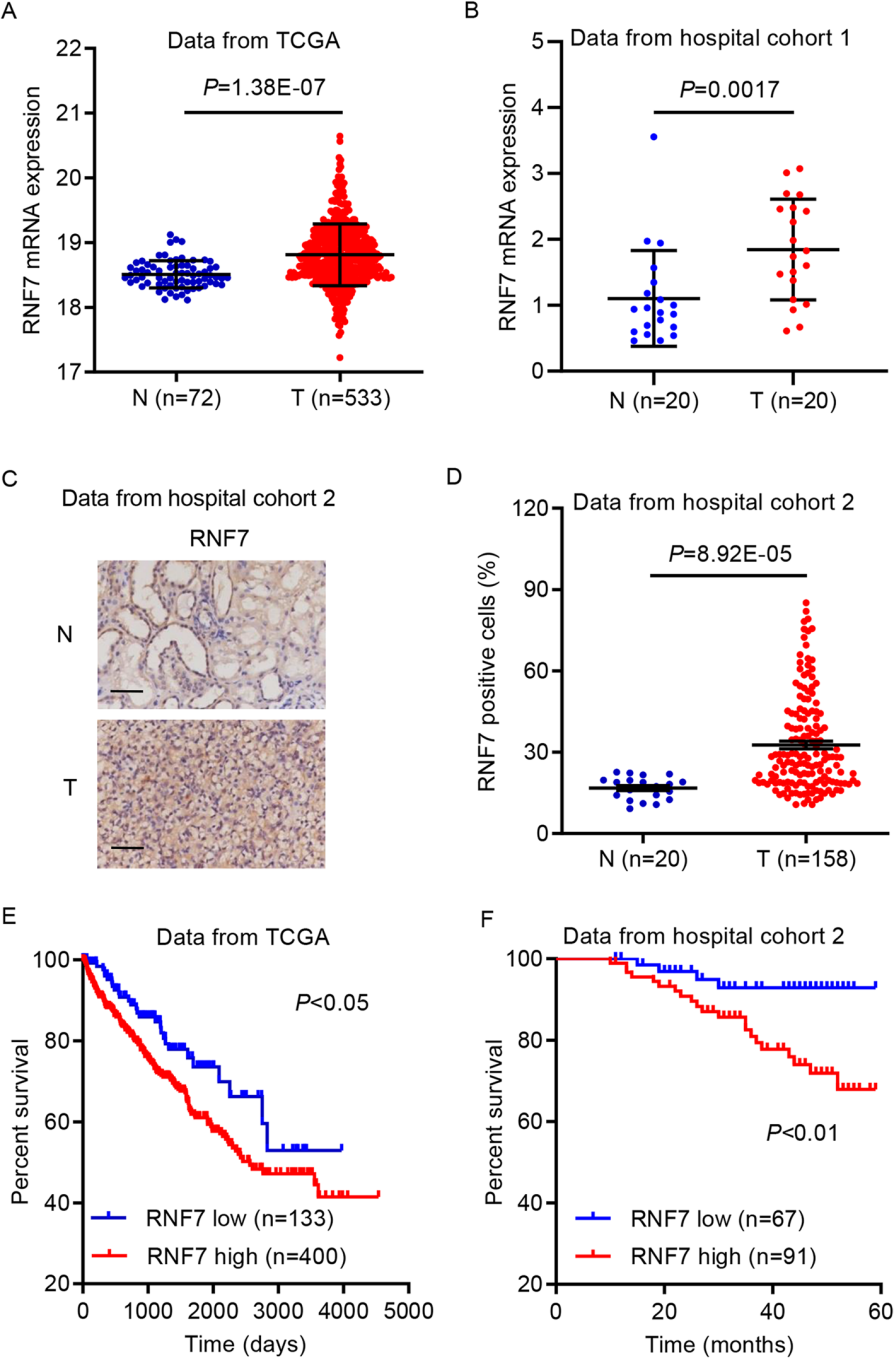
RNF7 expression is upregulated in ccRCC and is correlated with poor survival in patients with ccRCC. **A** RNF7 mRNA expression levels in ccRCC tissues (T, *n* = 533) and adjacent nontumor tissues (N, *n* = 72) from the TCGA database. **B** Comparison of RNF7 mRNA expression in ccRCC tissues (T, *n* = 20) and paired adjacent nontumor tissues (N, *n* = 20) from patients in hospital cohort 1. **C**, **D** Immunohistochemistry staining for RNF7 in ccRCC tissues (T, *n* = 158) and adjacent nontumor tissues (N, *n* = 20) from patients in hospital cohort 2. Scale bar: 50 µm. Survival probability of patients with ccRCC from (**E**) the TCGA database and (**F**) hospital cohort 2. Experiments performed in triplicate; data expressed as mean ± SD

First, the mRNA expression data of RNF7 in ccRCC from the TCGA database and hospital cohort 1 were analyzed. This analysis indicated that the mRNA expression levels of RNF7 were upregulated in ccRCC compared with those of nontumor adjacent tissues (Fig. 1A, B). IHC staining also revealed upregulated RNF7 expression in ccRCC tissues compared with nontumor adjacent tissues from hospital cohort 2 (Fig. 1C, D). It was also determined whether elevated RNF7 expression in ccRCC is clinically relevant. Using the hospital cohort 2 data, chi-square test was performed, which indicated that RNF7 expression was associated with tumor size, pathological T stage, grade, and American Joint Committee on Cancer (AJCC) stage, but not with any other clinicopathological features (Additional file 1: Table S1). Using the TCGA database (split patients by the low quartile) and hospital cohort 2 data, the relationship between RNF7 expression and overall survival rate in patients with ccRCC was examined. Interestingly, low RNF7 expression was significantly associated with high survival rate in patients with ccRCC (Fig. 1E, F). Univariate and multivariate analyses were performed to identify the prognostic factors of the overall survival using a Cox regression model (Additional file 1: Table S2). RNF7 expression levels, pathological T stage, and AJCC stage were identified as independent prognostic factors for ccRCC. Thus, RNF7 expression is a prognostic factor for ccRCC, and RNF7 upregulation is associated with poor patient survival.

### RNF7 knockdown enhances apoptosis and reduces glycolysis in RCC cell lines

**Fig. 2:**
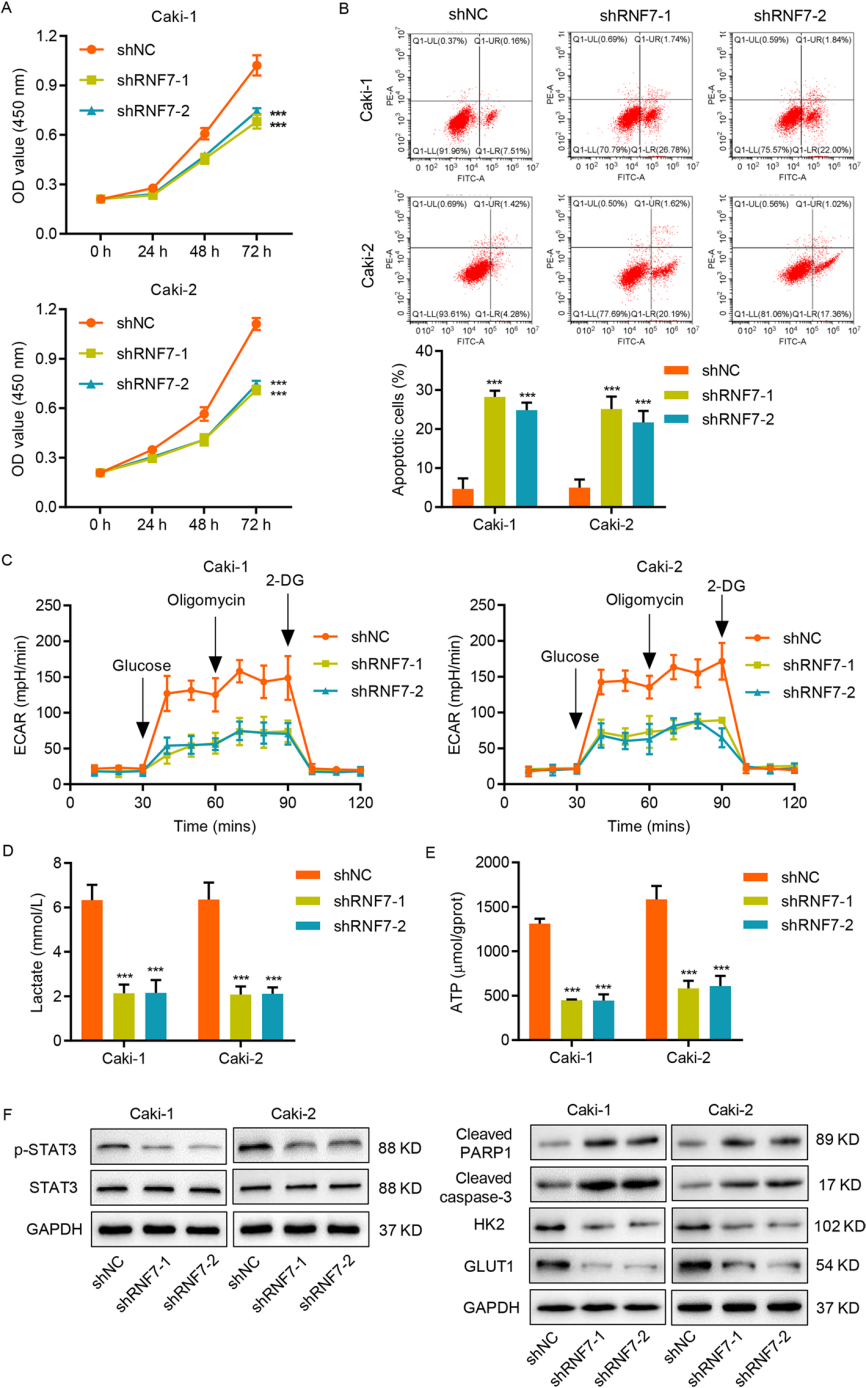
RNF7 knockdown enhances apoptosis and reduces glycolysis in RCC cell lines. Caki-1 and Caki-2 cells were transduced with RNF7 shRNAs (shRNF7-1 and shRNF7-2) or control scramble shRNA (shNC). **A** Cell viability was measured by CCK-8 assay. **B** Cell apoptosis and **C** ECAR level were analyzed by flow cytometry. **D** Lactate release and **E** ATP content were measured by biochemical analyses. **F** The protein levels of HK2, GLUT1, cleaved PARP1, cleaved caspase-3, STAT3, and p-STAT3 in Caki-1 and Caki-2 cells were determined by Western blot. Experiments performed in triplicate; data expressed as mean ± SD (*n* = 3). ***P < 0.001 compared with the shNC group

To determine how RNF7 contributes to RCC progression, the TCGA database was used to analyze the genes associated with RNF7 expression. GSEA revealed a strong correlation between RNF7 and apoptosis, glycolysis, and the JAK/STAT3 signaling pathway (Additional file 1: Fig. S1A). The notion that apoptosis influences malignant ccRCC phenotypes has been suggested [32], and alterations in metabolic pathways, such as glycolysis, play key roles in the survival of cancer cells [33]. To determine the relationship between these pathways and RNF7, shRNA-mediated knockdown of RNF7 in Caki-1 and Caki-2 cells was performed (Additional file 1: Fig. S1B-S1G). CCK-8 assay and flow cytometry analysis were conducted to assess the effects of RNF7 knockdown on cell viability and apoptosis, respectively. The results indicated thatRNF7 downregulation markedly reduced the cell viability and enhanced the apoptosis of Caki-1 and Caki-2 cells (Fig. 2A, B). To assess the effects of RNF7 on glycolysis, ECAR, lactate release, and adenosine triphosphate (ATP) levels were determined. The results indicated that RNF7 knockdown in Caki-1 and Caki-2 cells resulted in a decrease in ECAR levels (Fig. 2C), lactate release (Fig. 2D), and ATP content (Fig. 2E), suggesting that RNF7 downregulation led to reduced glycolysis. Consistent with these results, RNF7 knockdown also resulted in increased cleavage of PARP1 and caspase-3, decreased STAT3 phosphorylation, and decreased protein levels of STAT3 downstream targets, including HK2 [34] and GLUT1 [35] (Fig. 2F). These data demonstrate that RNF7 regulates apoptosis and glycolysis in RCC cells.

### RNF7 knockdown suppresses tumor growth in vivo

**Fig. 3:**
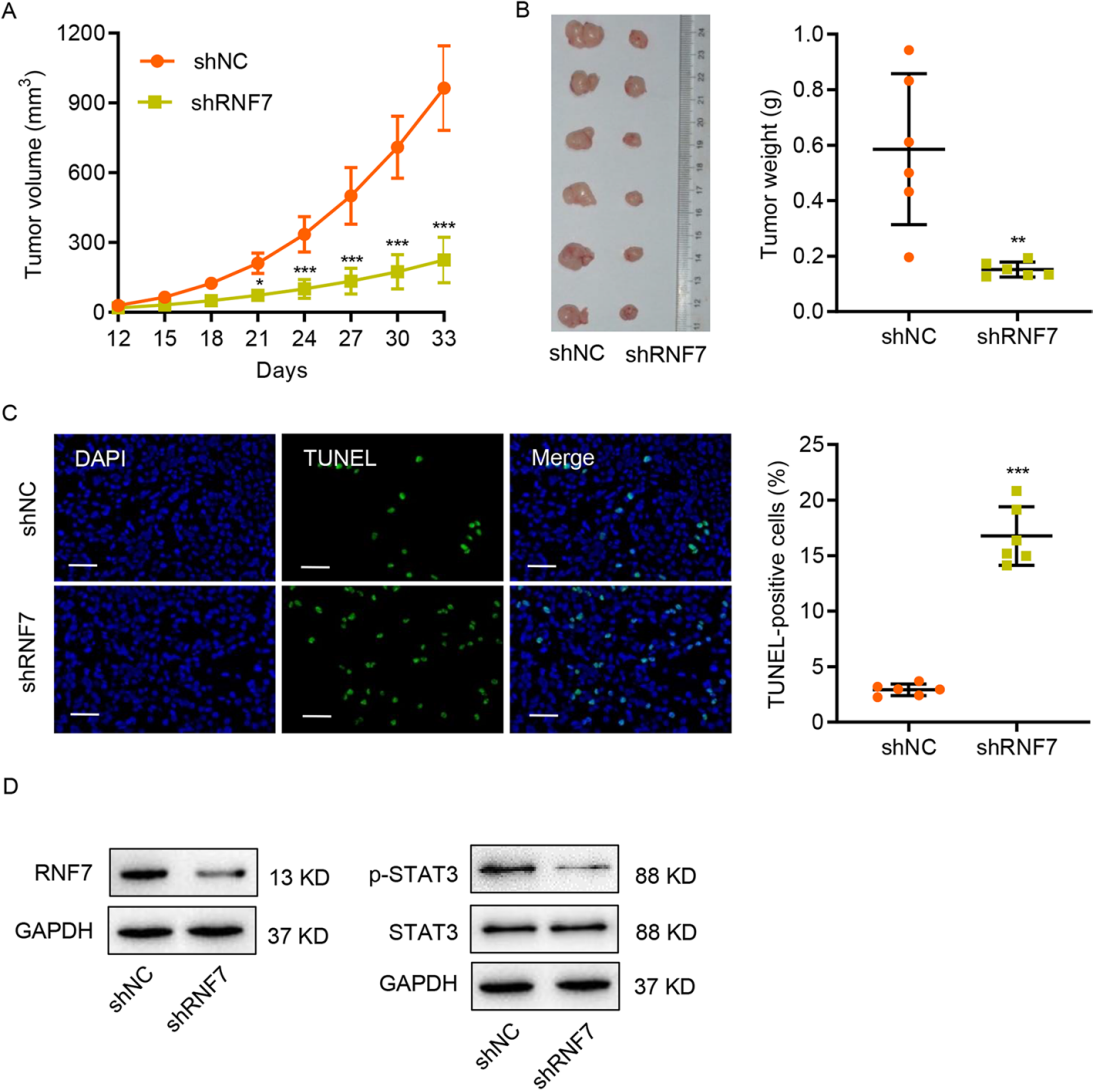
RNF7 knockdown inhibits tumor growth in vivo. Caki-1 cells were stably transduced with RNF7 shRNA or control scramble shRNA (shNC). Then, the cells were subcutaneously injected into nude mice (*n* = 6 per group). **A** Tumor volume was evaluated every 3 days for 33 days. **B** On day 33, the mice were sacrificed, and the tumors were photographed and weighed. **C** Representative images of TUNEL staining in xenograft mouse tumors. TUNEL-positive cells were quantified accordingly. Scale bar: 50 µm. **D** The protein levels of RNF7, STAT3, and p-STAT3 in xenograft mouse tumors were determined by Western blot. Experiments performed in triplicate; data expressed as mean ± SD (*n* = 6). *P < 0.05, **P < 0.01, ***P < 0.001 compared with shNC

To assess the importance of RNF7 in tumor growth in vivo, Caki-1 cells transduced with an RNF7 shRNA or control scramble shRNA were subcutaneously injected into nude mice. As shown in Fig. 3A–C, RNF7 knockdown dramatically inhibited the tumor growth and increased the apoptosis in vivo, suggesting that RNF7 has an oncogenic role. Moreover, decreased RNF7 expression and STAT3 phosphorylation level were observed in mouse xenograft tumors subjected to RNF7 knockdown (Fig. 3D). These findings suggest that RNF7 regulates tumor growth in vivo.

### RNF7 regulates apoptosis and glycolysis through the JAK/STAT3 signaling pathway

**Fig. 4:**
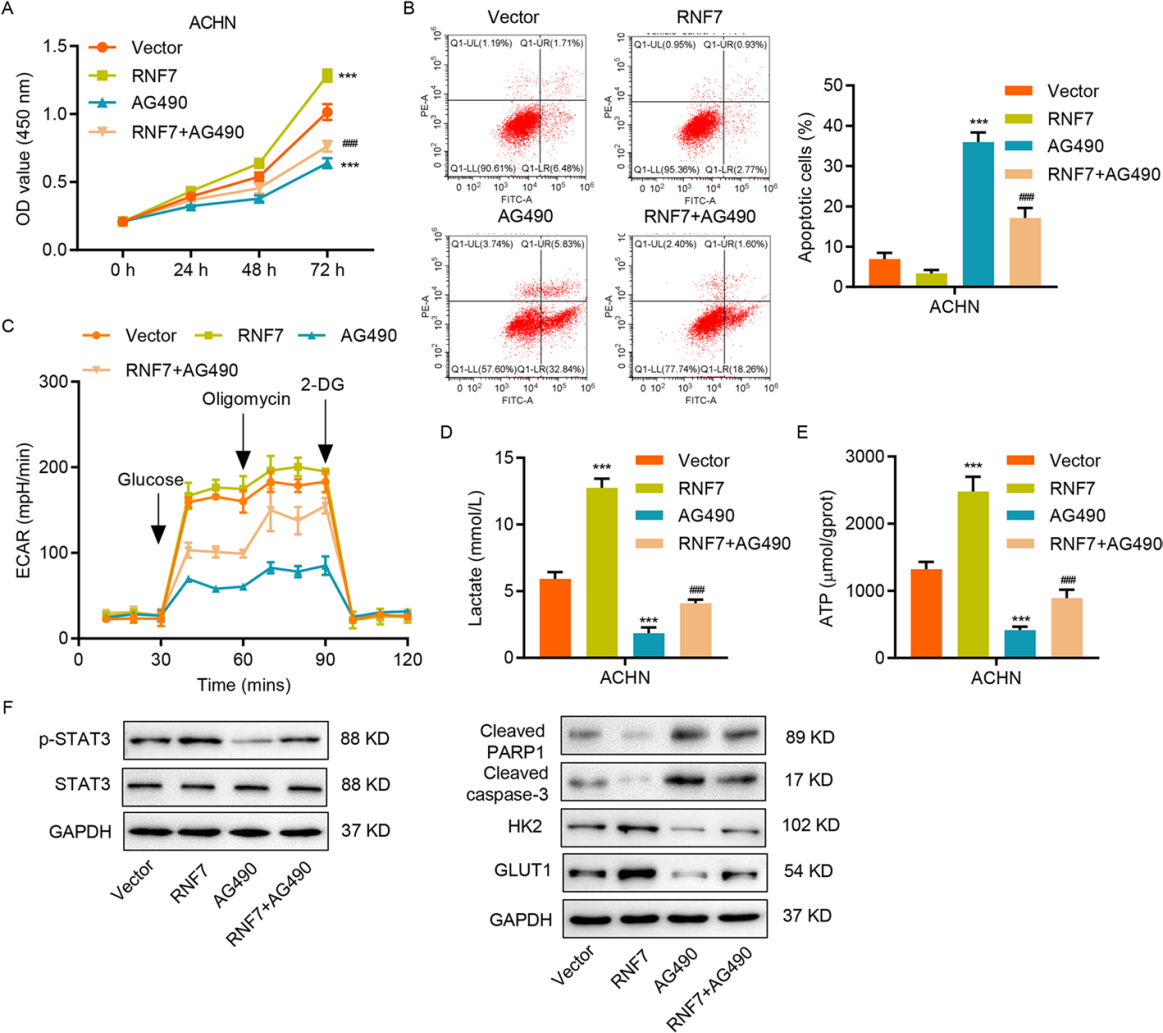
RNF7 overexpression reduces apoptosis and enhances glycolysis through the JAK/STAT3 signaling pathway. **A**–**F** ACHN cells transduced with RNF7-overexpressing lentivirus or blank lentivirus (vector) were treated with 10 µM AG490. **A** Cell viability was measured by CCK-8 assay. Flow cytometry was used to analyze **B** cell apoptosis and **D** ECAR level. **C** Lactate release and **E** ATP content were determined by biochemical analyses. **F** The protein levels of HK2, GLUT1, cleaved PARP1, cleaved caspase-3, STAT3, and p-STAT3 were determined by Western blot. Experiments performed in triplicate; data expressed as mean ± SD (*n* = 3). **P < 0.01, ***P < 0.001 compared with vector. ^###^*P* < 0.001 compared with RNF7

To determine whether RNF7 executes its effects on apoptosis and glycolysis via the JAK/STAT3 signaling pathway, RNF7 overexpression was performed in ACHN cells (Additional file 1: Fig. S1B-S1I). This significantly enhanced the cell viability, whereas the JAK2/3-specific inhibitor AG490 treatment strongly decreased it (Fig. 4A). Likewise, RNF7 induction markedly reduced the apoptosis of ACHN cells, whereas the AG490 treatment significantly increased the number of apoptotic cells (Fig. 4B). Of note, treating ACHN cells with AG490 suppressed the effects induced by RNF7 overexpression (Fig. 4A). Similar effects were observed in the regulation of glycolysis by the RNF7-JAK/STAT3 pathway (Fig. 4C–E). As shown in Fig. 4F, the AG490 treatment inhibited phosphorylation of STAT3, protein levels of HK2 and GLUT1, and cleavage of PARP1 and caspase-3. It also partially rescued the effects of RNF7 overexpression on cell viability, apoptosis, and glycolysis (Fig. 4A–F), suggesting that the effects of RNF7 are mediated, at least in part, by the JAK/STAT3signaling pathway.

### RNF7 interacts with and induces ubiquitination of SOCS1

**Fig. 5:**
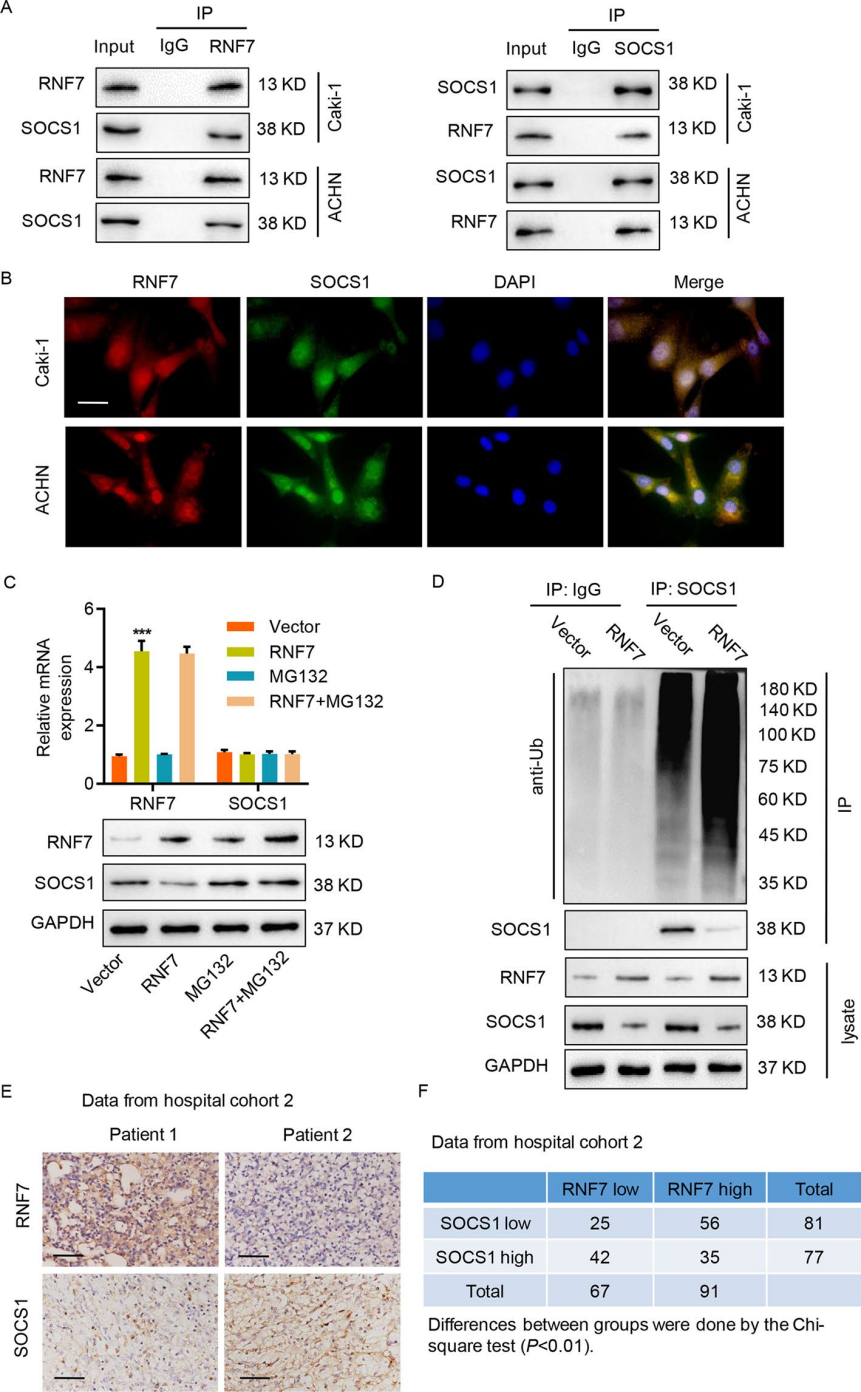
RNF7 interacts with and induces the ubiquitination of SOCS1. **A** Cell lysates were subjected to immunoprecipitation with control IgG, anti-RNF7, or anti-SOCS1 antibodies. Then, the immunoprecipitants were blotted with the indicated antibodies. **B** Immunofluorescence assay of RNF7 and SOCS1 in Caki-1 and ACHN cells. Scale bar: 20 µm. **C** ACHN cells were transduced with RNF7-overexpressing vector and subjected to MG132 (10 µM) treatment. The mRNA and protein expression levels of RNF7 and SOCS1 were determined by quantitative RT-PCR (top) and Western blot (bottom). **D** RNF7-overexpressing ACHN cells were immunoprecipitated with SOCS1 or IgG antibodies, and ubiquitination was evaluated by Western blot using anti-ubiquitin antibodies. **E** Immunohistochemistry staining for RNF7 and SOCS1 proteins in ccRCC tissues from patients in a hospital cohort. Scale bar: 50 µm. **F** Correlation study of RNF7 and SOCS1 in ccRCC tissues from patients in a hospital cohort. Experiments performed in triplicate; data expressed as mean ± SD (*n* = 3). ***P < 0.001 compared with vector

To elucidate the mechanisms by which RNF7 regulates JAK/STAT3 signaling, a co-immunoprecipitation experiment was performed using RNF7 antibodies, and its interaction with various JAK/STAT3 signaling proteins [36], including SHP-1, SHP-2, SOCS1, SOCS3, PTP1B, and PIAS1, was examined. As shown in Additional file 1: Fig. S2A, SOCS1 was specifically detected in RNF7-immunoprecipitated Caki-1 cell lysates by Western blot, but others, such as SHP-1, SHP-2, SOCS3, PTP1B, and PIAS1, did not show any interaction with RNF7. To verify the biochemical interaction between RNF7 and SOCS1, reciprocal co-immunoprecipitation assays in Caki-1 and ACHN cells were performed. As shown in Fig. 5A, immunoprecipitation with RNF7 and SOCS1 antibodies efficiently pulled down the respective proteins. Previous studies suggested that RNF7 acts as a component of E3 ubiquitin ligases [37], which regulate the degradation of various cellular proteins. RNF7 overexpression in ACHN cells did not alter the SOCS1 mRNA levels, but it did markedly reduce the SOCS1 protein levels (Fig. 5B). The immunofluorescence staining of RNF7 and SOCS1 also confirmed that RNF7 is co-localized with SOCS1 in Caki-1 and ACHN cells (Fig. 5B). Moreover, treatment with proteasome inhibitor MG132 in RNF7-overexpressing ACHN cells resulted in SOCS1 protein accumulation and rescuing of SOCS1 protein levels (Fig. 5C). To verify the regulation of SOCS1 ubiquitination by RNF7, RNF7-overexpressing ACHN cells were used to immunoprecipitate SOCS1, and Western blot was performed using anti-ubiquitin antibodies. As shown in Fig. 5D, RNF7 overexpression induced SOCS1 ubiquitination in ACHN cells, as detected by ubiquitin binding in SOCS1-immunoprecipitated protein lysates. Moreover, the MG132 treatment also increased the ubiquitination of SOCS1 induced by RNF7 overexpression and resulted in SOCS1 protein accumulation (Additional file 1: Fig. S2B). The immunohistochemistry staining of ccRCC tissues from tumor samples from patients in hospital cohort 2 also revealed expression of RNF7 and SOCS1 (Fig. 5E). The relationship between RNF7 and SOCS1 in ccRCC tissues collected from hospital cohort 2 was further confirmed. As shown in Fig. 5F, high RNF7 expression was significantly correlated with low SOCS1 expression in several ccRCC tissues, and several other tissues displayed low RNF7 expression and high SOCS1 expression.

### SOCS1 neutralizes RNF7-mediated effects on apoptosis and glycolysis in RCC cell lines

**Fig. 6:**
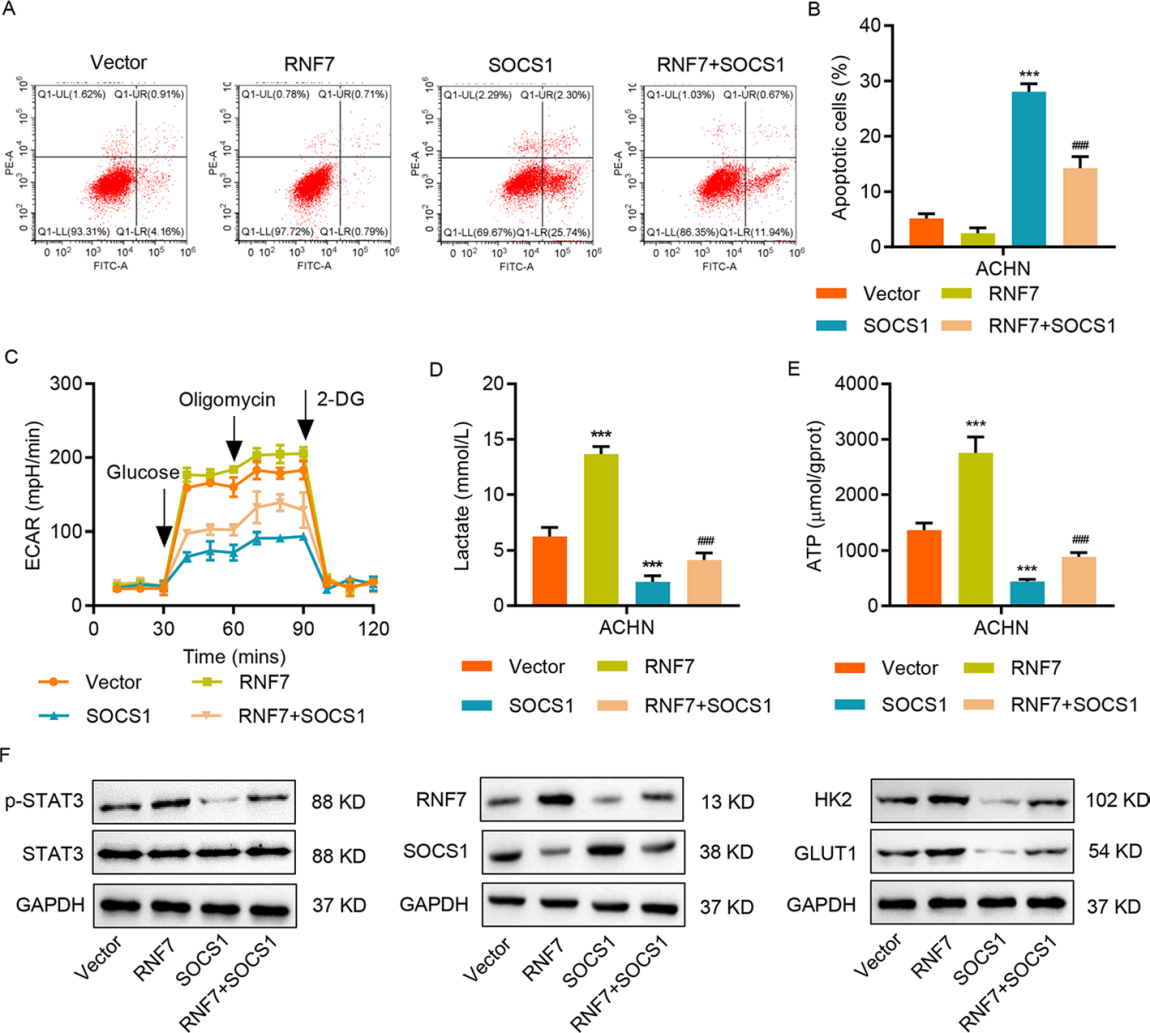
SOCS1 neutralizes the RNF7-mediated effects on apoptosis and glycolysis in ccRCC cell lines. ACHN cells were transduced with the indicated plasmids, and flow cytometry was performed to assess (**A**, **B**) cell apoptosis and **C** ECAR level. Biochemical assays were performed to measure **D** lactate release and **E** ATP content. **F** The protein levels of RNF7, SOCS1, HK-2, GLUT1, STAT3, and p-STAT3 were determined by Western blot. Experiments performed in triplicate; data expressed as mean ± SD (*n* = 3). ***P < 0.001 compared with vector. ^###^*P* < 0.001 compared with RNF7

Functionally, OCS1 overexpression (Additional file 1: Fig. S2C) antagonized the RNF-induced decrease in apoptosis (Fig. 6A, B) and suppressed the RNF7-induced increase in glycolysis, lactate release, and ATP content (Fig. 6C–E), suggesting that the SOCS1/JAK/STAT3 pathway mediates the downstream functions of RNF7. Consistent with these findings, RNF7 overexpression induced STAT3 phosphorylation, whereas SOCS1 overexpression displayed the opposite regulation and inhibited JAK/STAT3 signaling (Fig. 6F).

### RNF7 regulates sensitivity to sunitinib in ccRCC cells

**Fig. 7:**
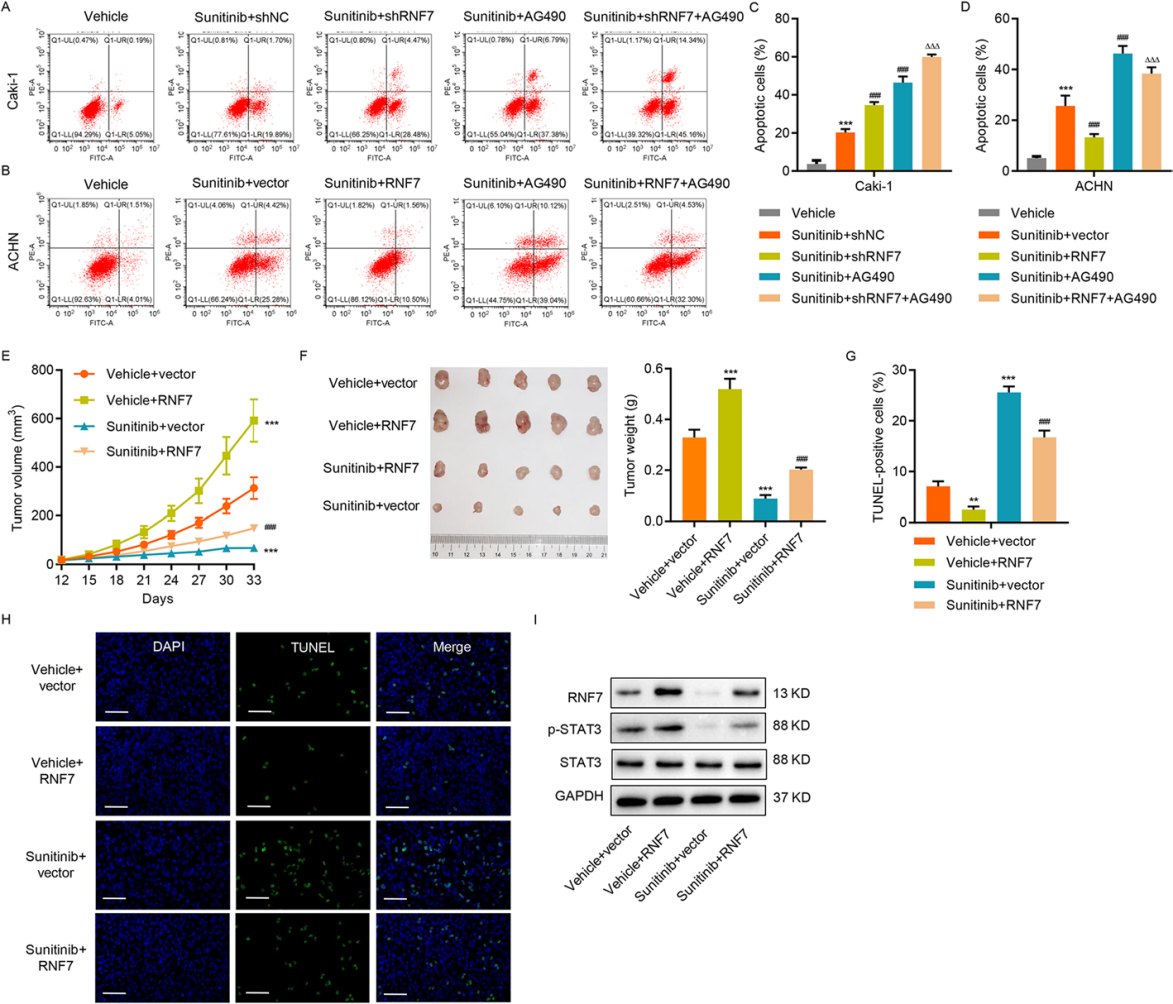
RNF7 regulates the sensitivity of ccRCC cells to sunitinib. **A**, **C** Caki-1 cells transduced with RNF7 shRNA or control scramble shRNA (shNC) and **B**, **D** ACHN cells transduced with RNF7-overexpressing lentivirus or blank lentivirus (vector) were treated with sunitinib (1 μM) or vehicle in absence or presence of 10 µM AG490 for 24 h, and cell apoptosis was subsequently measured (*n* = 3 per group). ACHN cells were stably transduced with RNF7-overexpressing lentivirus or blank lentivirus (vector), then these cells were subcutaneously injected into nude mice (*n* = 5 per group). After 12 days, the mice received an intraperitoneal injection of sunitinib (20 mg/kg) every other day. On day 33, the **E** tumor volume and **F** weight were measured. **G**, **H** Xenograft tumors with TUNEL staining. Scale bar: 50 µm. **I** The protein levels of RNF7, STAT3, and p-STAT3 in xenograft mouse tumors were determined by Western blot. Experiments performed in triplicate; data expressed as mean ± SD (*n* = 3 or 5). **P < 0.01, ***P < 0.001 compared with vehicle or vehicle + vector. ^###^*P* < 0.001 compared with sunitinib + shNC or sunitinib + vector. ^ΔΔΔ^*P* < 0.001 compared with sunitinib + shRNF7 or sunitinib + RNF7

The potential clinical relevance of these findings was investigated by researching the effects of RNF7 on the sensitivity of ccRCC cells to sunitinib, a tyrosine kinase receptor inhibitor targeting vascular endothelial growth factor receptors (VEGFRs) and platelet-derived growth factor receptors (PDGFRs) [38]. The knockdown of RNF7 in Caki-1 cells increased the sunitinib sensitivity and dramatically induced apoptosis (Fig. 7A, C), whereas RNF7 overexpression in ACHN cells decreased the sunitinib sensitivity and significantly diminished apoptosis (Fig. 7B, D). When combined with RNF7 knockdown, the AG490 treatment augmented the effects of RNF7 knockdown on apoptosis induction, whereas the AG490 treatment inRNF7-overexpressing cells suppressed the RNF7 overexpression-induced effects. These results demonstrate that RNF7 influences the sensitivity of ccRCC to sunitinib via regulation of JAK/STAT3 signaling. The role of RNF7 in modulating sunitinib sensitivity was further tested in vivo by subcutaneously injecting RNF7-overexpressing ACHN cells into nude mice. Interestingly, sunitinib treatment in vector-expressing ACHN cells significantly reduced the tumor volume and weight (Fig. 7E, F). However, sustained RNF7 overexpression inhibited the sunitinib-induced effects (Fig. 7E, F), suggesting that RNF7 expression is crucial in the regulation of sunitinib sensitivity in ccRCC cells. Finally, the effects of RNF7 on sunitinib sensitivity in ACHN cells were highly correlated with the potency of RNF7 in reducing apoptosis (Fig. 7G, H). Moreover, increased RNF7 and p-STAT3 levels were also observed in mouse xenograft tumors after RNF7 overexpression, whereas these were decreased after the sunitinib treatment (Fig. 7I). These results provide supporting evidence that RNF7 plays a role in regulating the sensitivity of ccRCC to sunitinib, therefore highlighting the importance of targeting this protein in developing a highly efficient strategy for killing ccRCC cells.

### STAT3 activation transcriptionally regulates RNF7 expression

**Fig. 8:**
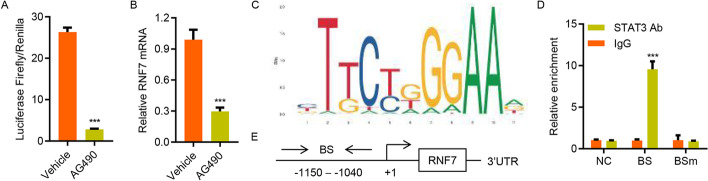
STAT3 activation transcriptionally regulates the expression of RNF7. **A** A luciferase reporter assay was performed to evaluate the activity of the RNF7 promoter in Caki-1 cells treated with AG490 or vehicles. **B** Quantitative RT-PCR analysis of the effects of AG490 (10 μM) on RNF7 mRNA expression. **C** The STAT3 binding site in the RNF7 promoter was predicted by the JASPAR algorithm. **D** The ChIP assay results indicate that STAT3 bound to the RNF7 promoter. **E** Schematic diagram of the primers used in the ChIP analysis. 3′-UTR served as a negative control (NC). BS, binding site; BSm, mutant binding site. Experiments performed in triplicate; data expressed as mean ± SD (*n* = 3). ***P < 0.001 compared with vehicle or IgG

Finally, the mechanism underlying the increase in RNF7 expression in ccRCC was investigated. Because STAT3 regulates gene transcription, we examined whether STAT3 affects the activity of the RNF7 promoter. As illustrated in Fig. 8A, B, the relative luciferase activity of the RNF7 promoter and the mRNA expression level of RNF7 were markedly decreased in AG490-treated Caki-1 cells. Using JASPAR (http://jaspar.genereg.net/), a high-quality transcription factor binding profile database, a potential STAT3 binding site in the RNF7 promoter was identified (Fig. 8C). ChIP assay was performed on Caki-1 cells to confirm that STAT3 was directly bound to the RNF7 promoter. As shown in Fig. 8D, E, STAT3 was bound to the RNF7 promoter but not to 3′-untranslated region (UTR) or mutant site, suggesting that it shows direct regulation of RNF7 via a feedback loop mechanism.

## Discussion

In this study, the role of RNF7, a RING finger protein and component of the protein degradation machinery, in RCC was investigated. By analyzing the RNF7 mRNA expression data from the TCGA database, along with tumor tissues from the hospital cohort of patients with ccRCC, we found that RNF7 was upregulated in ccRCC tissues and was correlated with poor survival. A similar pattern of upregulated expression was observed in RCC cell lines compared with normal human proximal renal tubular HK-2 cells. Through gene enrichment analyses, functional assays, and biochemical experiments, a RNF7-SOCS1/JAK/STAT3 pathway was uncovered, and its functional relevance in regulating cell viability, apoptosis, and glycolysis in vitro and tumor growth in vivo was demonstrated (Additional file 1: Fig. S3).

Accumulating evidence also indicates that RNF7 plays a key role in several other human cancers. In prostate cancer, high RNF7 expression is known to influence tumor progression, and RNF7 knockdown enhances the sensitivity of prostate cancer cells to cisplatin [17]. Similarly, our findings indicate that altering the RNF7 expression in ccRCC cells affects the apoptosis and strongly influences the response of ccRCC cells to sunitinib. Hence, targeting RNF7 may have broad implications in cancer therapy.

This study revealed the important functions of the RNF7-JAK/STAT3 pathway in modulating apoptosis and glycolysis, which are utilized by cancer cells to escape multiple levels of stress imposed by the immune system and various drugs [39, 40]. STAT3 is important in tumor cell survival, and there is growing interest in targeting STAT3 to induce cancer cell apoptosis [41]. STAT3 has also been shown to potentiate glucose metabolism and accelerate glycolysis to support malignant ccRCC phenotypes [42]. Thus, the RNF7-JAK/STAT3 pathway is an attractive target for ccRCC treatment. AG490 is a tyrosine kinase inhibitor that has been extensively used for inhibiting JAK2/3 as a method of blocking STAT3 activation in vitro and in vivo [43, 44]. The reversal of the effects of RNF7 overexpression by JAK/STAT3 pathway inhibition via AG490 treatment indicates that JAK/STAT3 signaling is a crucial mediator of the downstream functions of RNF7. However, to further verify that the effects of RNF7 are mediated by the STAT3 signaling pathway, experiments with genetic STAT3 inhibition, such as STAT3 knockdown, should be provided in RCC cell lines. Moreover, the lack of a more specific STAT3 inhibitor such as Stattic is also a limitation of this study, and experiments need to be repeated with Stattic to verify that the effects of RNF7 are mediated by the STAT3 signaling pathway.

Mechanistically, RNF7 activates the JAK/STAT3 pathway by interacting with and inducing SOCS1 ubiquitination, which acts as a negative regulator of JAK/STAT3 signaling by inhibiting JAK kinase activities [45]. This regulation is functionally relevant because SOCS1 overexpression blocked the RNF7-mediated effects on STAT3 signaling and STAT3-mediated effects on cell viability, apoptosis, and glycolysis. The identification of a regulatory pathway from RNF7 to SOCS1/JAK/STAT3 is particularly exciting because STAT3 activation is widely observed in several human cancers [21, 46]. In fact, efforts to identify STAT3-targeting compounds have been actively pursued [22, 47]. Given the reported roles of STAT3 in cancers, in which RNF7 overexpression has also been observed [41, 48, 49], it will be interesting to examine whether the regulation of SOCS1/JAK/STAT3 by RNF7 also operates in other cancer types.

One of the key findings of this study is the role of RNF7 in reducing the sensitivity of ccRCC cells to sunitinib treatment. This has high clinical relevance because eliminating the viability of drug-resistant cancer cells remains a crucial challenge in cancer treatment. In the past decade, sunitinib has shown success as a first-line therapy for advanced RCC. Clinically, it has been shown that sunitinib is associated with higher treatment response rates, longer progression-free survival, and decreased toxicity [50]. RNF7 also strongly influences the response and sensitivity of ccRCC cells to sunitinib. It has also been shown independently that STAT3 inhibition induces RCC tumor cell apoptosis and reduces the number of immunosuppressive cells [24]. Our findings reveal an additional layer of regulatory mechanism and demonstrate a previously unknown link between RNF7 and JAK/STAT3. These findings also highlight the potential of combining the RNF7-JAK/STAT3-targeting approach with sunitinib treatment to efficiently kill resistant cancer cells. However, the specificity of RNF7 knockdown, the effects on STAT3 activity, and a triple combination therapy of sunitinib, STAT3 inhibition, and RNF7 knockdown on regulating tumor growth in vivo need to be investigated further.

Finally, this study demonstrated that STAT3 directly regulates the RNF7 promoter. This feedback loop mechanism is an attractive target for cancer treatment, as shutting down this regulatory signaling can have a marked effect on cancer cell survival. Precisely how RNF7 contributes to ccRCC progression and whether there are additional targets downstream of RNF7 remain important issues that need to be elucidated in future investigations. How RNF7 modulates the response of ccRCC cells to sunitinib and other cancer drugs also need to be elucidated. Given the functions of RNF7-SOCS1/STAT3 in regulating cell survival and apoptosis, it will be interesting to evaluate their effects on cell cycle progression and DNA repair, which have been suggested to influence the drug response [51–53]. Future investigations on these potential mechanisms are crucial to gain insight into how RNF7 regulates the sensitivity of cancer cells to treatments.

## Conclusions

RNF7 regulates apoptosis, glycolysis, and sunitinib sensitivity in ccRCC via the SOCS1/JAK/STAT3 signaling pathway, suggesting that RNF7 may serve as a prognostic biomarker and drug target in ccRCC management. These observations also provide new evidence for an interplay between cancer-associated signaling pathways and the regulation of apoptosis, glycolysis, and sunitinib sensitivity.

## Supplementary Information

**Additional file 1: Table S1.** Relationship between RNF7 expression and clinicopathological features of clear cell renal cell carcinoma. **Table S2.** Univariate and multivariate analyses of overall survival in patients with clear cell renal cell carcinoma. **Figure S1**. GSEA and expression of RNF7 and SOCS1 in Caki-1, Caki-2, and ACHN cells. (A) GSEA indicated that RNF7 expression in ccRCC tissues from the TCGA database correlated with genes involved in apoptosis, glycolysis, and JAK/STAT3 signaling pathways. (B–D) RNF7 mRNA and protein expression levels in RCC cell lines (786-O, A498, ACHN, Caki-1, and Caki-2) and human proximal tubular HK-2 cells. (E–G) mRNA and protein levels of RNF7 in Caki-1 and Caki-2 cells transduced with RNF7 shRNAs (shRNF7-1, shRNF7-2, and shRNF7-3) or control scramble shRNA (shNC). (H, I) mRNA and protein levels of RNF7 in ACHN cells transduced with RNF7-overexpressing lentivirus or blank lentivirus (vector). Experiments performed in triplicate; data expressed as mean ± SD (*n* = 3). ***P < 0.001 compared with shNC or vector. **Figure S2**. RNF7 interacts with ubiquitinates and destabilizes SOCS1. (A) Caki-1 cell lysates were subjected to immunoprecipitation with control IgG, anti-RNF7, anti-SHP-1, anti-SHP-2, anti-SOCS1, anti-SOCS3, or anti-PTP1B antibodies. Then, the immunoprecipitants were blotted with the indicated antibodies. (B) RNF7-overexpressing ACHN cells treated with or without MG132 were immunoprecipitated with SOCS1 antibodies, and ubiquitination and expression were evaluated by Western blot using anti-ubiquitin and anti-SOCS1 antibodies, respectively. (C) mRNA (upper) and protein (bottom) levels of SOCS1 in ACHN cells transduced with RNF7-overexpressing lentivirus, SOCS1-overexpressing lentivirus, or blank lentivirus (vector). Experiments performed in triplicate; data expressed as mean ± SD (*n* = 3). ***P < 0.001 compared with vector. **Figure S3**. Schematic representation of RNF7 in the regulation of apoptosis, glycolysis, and sunitinib sensitivity in renal cell carcinoma via the SOCS1/JAK/STAT3 feedback loop.

## Data Availability

All data presented in this study are included within the paper and its Additional files.

## Abbreviations

RNF7: RING finger protein 7
RCC: Renal cell carcinoma
ccRCC: Clear cell RCC
VHL: von Hippel–Lindau
VEGF: Vascular endothelial growth factor
PDGF: Platelet-derived growth factor
STAT3: Signal transducer and activator of transcription 3
SOCS: Suppressors of cytokine signaling
IHC: Immunohistochemistry
CCK-8: Cell counting kit-8
ECAR: Extracellular acidification rate
ChIP: Chromatin immunoprecipitation
RT-PCR: Reverse-transcription PCR
TUNEL: Terminal deoxynucleotidyl transferase dUTP nick end labeling
