# Supplementary material for: RNF7 inhibits apoptosis and sunitinib sensitivity and promotes glycolysis in renal cell carcinoma via the SOCS1/JAK/STAT3 feedback loop
Source: Cell Mol Biol Lett. 2022 May 13;27:36. doi: 10.1186/s11658-022-00337-5 (PMC9107170; doi:10.1186/s11658-022-00337-5)
Supplement: Supplementary file 1 — Additional file 1: Table S1. Relationship between RNF7 expression and clinicopathological features of clear cell renal cell carcinoma. Table S2. Univariate and multivariate analyses of overall survival in patients with clear cell renal cell carcinoma. Figure S1. GSEA and expression of RNF7 and SOCS1 in Caki-1, Caki-2, and ACHN cells. (A) GSEA indicated that RNF7 expression in ccRCC tissues from the TCGA database correlated with genes involved in apoptosis, glycolysis, and JAK/STAT3 signaling pathways. (B–D) RNF7 mRNA and protein expression levels in RCC cell lines (786-O, A498, ACHN, Caki-1, and Caki-2) and human proximal tubular HK-2 cells. (E–G) mRNA and protein levels of RNF7 in Caki-1 and Caki-2 cells transduced with RNF7 shRNAs (shRNF7-1, shRNF7-2, and shRNF7-3) or control scramble shRNA (shNC). (H, I) mRNA and protein levels of RNF7 in ACHN cells transduced with RNF7-overexpressing lentivirus or blank lentivirus (vector). Experiments performed in triplicate; data expressed as mean ± SD (n = 3). ***P < 0.001 compared with shNC or vector. Figure S2. RNF7 interacts with ubiquitinates and destabilizes SOCS1. (A) Caki-1 cell lysates were subjected to immunoprecipitation with control IgG, anti-RNF7, anti-SHP-1, anti-SHP-2, anti-SOCS1, anti-SOCS3, or anti-PTP1B antibodies. Then, the immunoprecipitants were blotted with the indicated antibodies. (B) RNF7-overexpressing ACHN cells treated with or without MG132 were immunoprecipitated with SOCS1 antibodies, and ubiquitination and expression were evaluated by Western blot using anti-ubiquitin and anti-SOCS1 antibodies, respectively. (C) mRNA (upper) and protein (bottom) levels of SOCS1 in ACHN cells transduced with RNF7-overexpressing lentivirus, SOCS1-overexpressing lentivirus, or blank lentivirus (vector). Experiments performed in triplicate; data expressed as mean ± SD (n = 3). ***P < 0.001 compared with vector. Figure S3. Schematic representation of RNF7 in the regulation of apoptosis, glycolysis, and sunitinib sensi [file 11658_2022_337_MOESM1_ESM.docx]

**Table S1.** Relationship between RNF7 expression and clinicopathological features of clear cell renal cell carcinoma

| Clinicopathological features | RNF7 | | *P* value |
| --- | --- | --- | --- |
|  | Low (n=67) | High (n=91) |  |
| **Gender** |  |  | 0.4099 |
| Male (n=110) | 49 | 61 |  |
| Female (n=48) | 18 | 30 |  |
| **Age (years)** |  |  | 0.2428 |
| ≤60 (n=84) | 32 | 52 |  |
| >60 (n=74) | 35 | 39 |  |
| **Tumor size (cm)** |  |  | 0.0028 |
| ≤3 (n=30) | 20 | 10 |  |
| >3 (n=128) | 47 | 81 |  |
| **T**  T1+T2 (n=144)  T3+T4 (n=14) | 65  2 | 79  12 | 0.0257 |
| **N**  N0 (n=156)  N1 (n=2) | 67  0 | 89  2 | 0.2220 |
| **M**  M0 (n=154)  M1 (n=4) | 67  0 | 87  4 | 0.0822 |
| **Grade**  I+II (n=128)  III+IV (n=30) | 60  7 | 68  23 | 0.0189 |
| **AJCC stage** |  |  | 0.0130 |
| 1+2 (n=146) | 66 | 80 |  |
| 3+4 (n=12) | 1 | 11 |  |

Differences between groups were done by the Chi-square test.

**Table S2.** Univariate and multivariate analysis of overall survival in patients with clear cell renal cell carcinoma

| Variables | Univariate analysis | | Multivariate analysis | |
| --- | --- | --- | --- | --- |
|  | HR (95% CI) | *P* | HR (95% CI) | *P* |
| Age (≤60 vs >60) | 1.162 (0.9411-1.458) | 0.1627 |  |  |
| Gender (Female vs Male) | 0.8491 (0.6966-1.066) | 0.1461 |  |  |
| Tumor size (cm) (>3 vs ≤3) | 1.270 (0.9889-1.526) | 0.0591 |  |  |
| T (T3+T4 vs T1+T2) | 5.201 (1.848-18.58) | <0.001 | 2.022 (1.163-4.443) | 0.0048 |
| N (N1 vs N0) | 1.385 (0.749-7.339) | 0.5591 |  |  |
| M (M1 vs M0) | 2.805 (0.9918-15.41) | 0.0540 |  |  |
| Grade (III+IV vs I+II) | 1.590 (1.137-2.480) | 0.0033 |  |  |
| AJCC stage (3+4 vs 1+2) | 2.158 (1.165-5.232) | 0.0055 | 1.710 (1.029-3.707) | 0.0333 |
| RNF7 expression level (high vs low) | 1.383 (1.129-1.712) | 0.0022 | 1.285 (1.045-1.587) | 0.0183 |


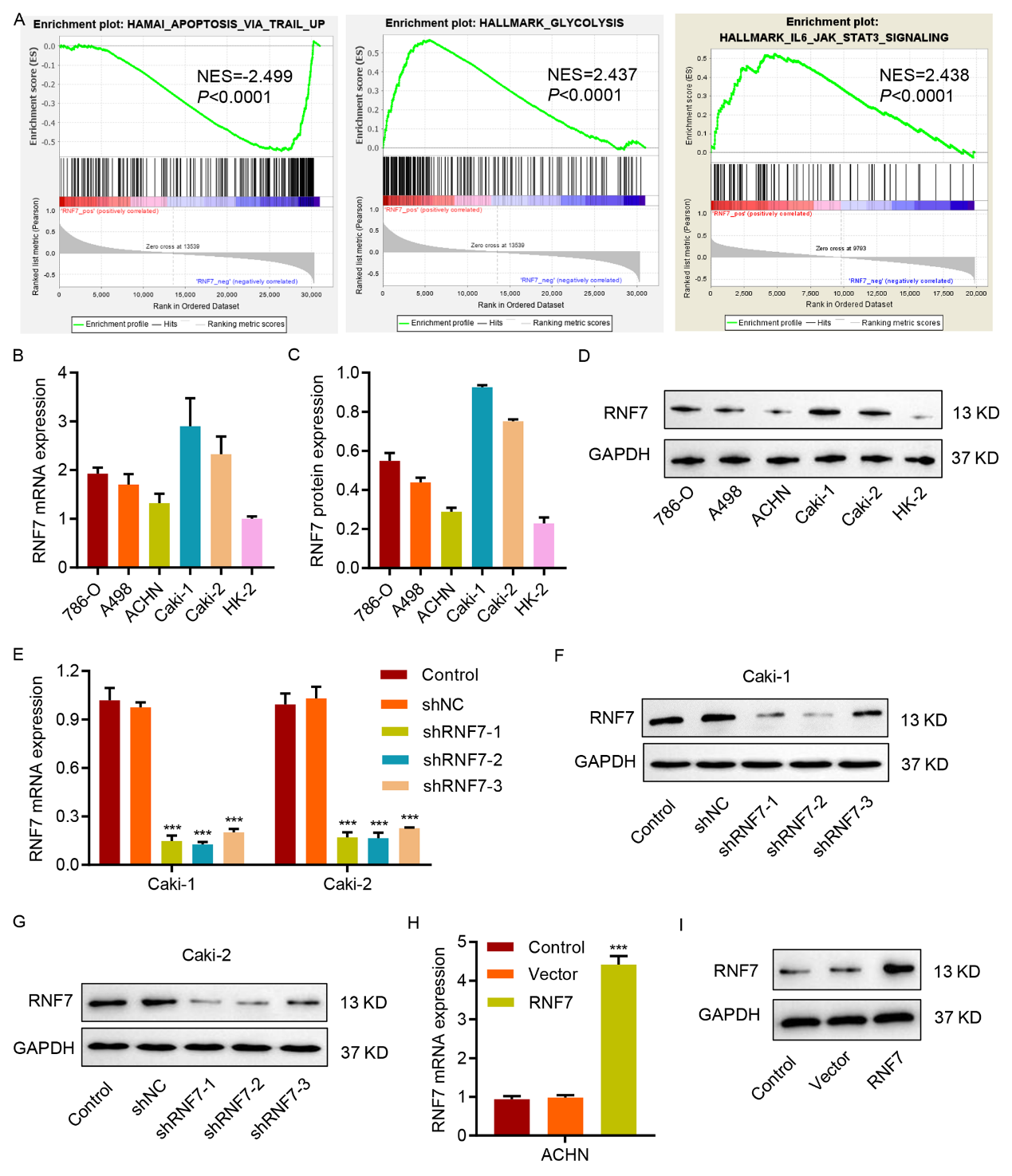


**Figure S1. GSEA and expression of RNF7 and SOCS1 in Caki-1, Caki-2, and ACHN cells.** (A) GSEA indicated that RNF7 expression in ccRCC tissues from the TCGA database correlated with genes involved in apoptosis, glycolysis, and JAK/STAT3 signaling pathways. (B–D) RNF7 mRNA and protein expression levels in RCC cell lines (786-O, A498, ACHN, Caki-1, and Caki-2) and human proximal tubular HK-2 cells. (E–G) mRNA and protein levels of RNF7 in Caki-1 and Caki-2 cells transduced with RNF7 shRNAs (shRNF7-1, shRNF7-2 and shRNF7-3) or control scramble shRNA (shNC). (H, I) mRNA and protein levels of RNF7 in ACHN cells transduced with RNF7-overexpressing lentivirus or blank lentivirus (vector). Experiments were performed in triplicate, and the data are expressed as mean + SD (n = 3). ****P*<0.001 compared with shNC or vector.


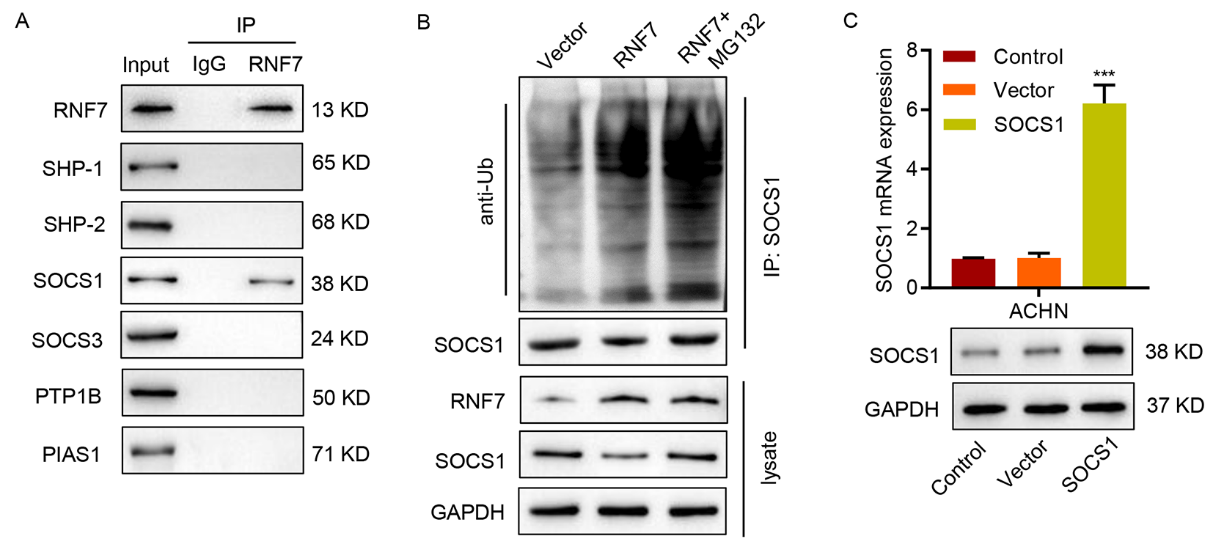


**Figure S2. RNF7 interacts with ubiquitinates and destabilizes SOCS1.** (A) Caki-1 cell lysates were subjected to immunoprecipitation with control IgG, anti-RNF7, anti-SHP-1, anti-SHP-2, anti-SOCS1, anti-SOCS3, or anti-PTP1B antibodies. Then, the immunoprecipitants were blotted with the indicated antibodies. (B) RNF7-overexpressing ACHN cells treated with or without MG132 were immunoprecipitated with SOCS1 antibodies, and ubiquitination and expression were evaluated by Western blot using anti-ubiquitin and anti-SOCS1 antibodies, respectively. (C) mRNA (upper) and protein (bottom) levels of SOCS1 in ACHN cells transduced with RNF7-overexpressing lentivirus, SOCS1-overexpressing lentivirus, or blank lentivirus (vector). Experiments were performed in triplicate, and the data are expressed as mean + SD (n = 3). ****P*<0.001 compared with vector.


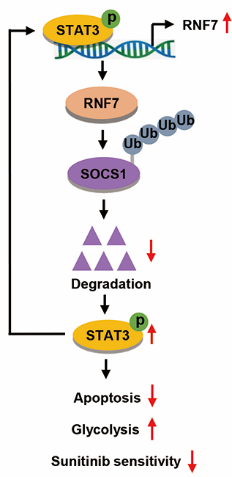


**Figure S3.** Schematic representation of RNF7 in the regulation of apoptosis, glycolysis, and sunitinib sensitivity in renal cell carcinoma via the SOCS1/JAK/STAT3 feedback loop.
